# Management of Heart Failure With Preserved Ejection Fraction in Elderly Patients: Effectiveness and Safety

**DOI:** 10.7759/cureus.35030

**Published:** 2023-02-15

**Authors:** Amr Elkammash, Simpson Shiu Chung Tam, Geethana Yogarajah, Jianing You

**Affiliations:** 1 Department of Cardiology, The Royal Papworth Hospital NHS Foundation Trust, Cambridge, GBR; 2 School of Clinical Medicine, University of Cambridge, Cambridge, GBR

**Keywords:** renin-angiotensin-aldosterone system (raas), side-effects, mineralocorticoid receptor antagonist, beta-blockers, sacubitril-valsartan, cardiomems, sodium-glucose cotransporter 2 (sglt-2) inhibitors, elderly, heart failure with preserved ejection fraction

## Abstract

The proportion of the elderly population continues to increase due to the global increase in longevity. Heart failure with preserved ejection fraction (HFpEF) is common in the elderly due to cellular aging, myocardial stiffness, and multiple comorbidities. This age group is often under-represented in clinical trials. In this narrative review, we looked into the latest evidence-based lines of management of HFpEF in this vulnerable cohort. In this narrative review, we brought the latest evidence on the treatment of HFpEf in the elderly. We searched the largest three scientific databases (Pubmed, Google Scholar, and EMBASE) using the search words (elderly, HFpEF, heart failure with preserved ejection fraction, guidelines, treatment, and management) in different combinations. To date, screening for and treatment of the causes of HFpEF (such as hypertension, coronary artery disease [CAD], valvular heart disease, and cardiac amyloidosis) and associated comorbidities (such as diabetes mellitus [DM], iron deficiency, obesity, and thyroid dysfunction) are the main line of management of HFpEF. A multidisciplinary team, including an HF specialist cardiologist, an HF nurse, a geriatrician, a dietician, a psychologist, a physiotherapist, and an occupational therapist, should manage HFpEF elderly patients. Other specialist input may be needed according to the patient's requirements. The evidence on the effective management of HFpEF in the elderly age group is scarce and controversial. Some studied non-pharmacological approaches include supervised exercise training, pulmonary artery pressure monitoring, and the interatrial shunt device (an emerging modality that includes a small percutaneously inserted interatrial left to right valve aiming to reduce the left atrial and pulmonary wedge pressures). These modalities can only improve the symptoms and HF hospitalizations without robustly impacting cardiovascular (CV) death. Among the pharmacological approaches to treat HFpEF, only the sodium-glucose cotransporter 2 (SGLT-2) inhibitors proved efficacy in reducing the hard outcomes of CV death, HF hospitalizations, and urgent visits for HF when used in elderly HFpEF patients, irrespective of the presence of diabetes mellitus. Diuretics are only beneficial to alleviate the symptoms of fluid overload, with a risk of renal impairment in volume-depleted patients. The evidence on the effectiveness of other HF-specific disease-modifying agents in elderly HFpEF patients is controversial. Elderly patients have a higher risk of having side effects from HF medications due to the higher prevalence of polypharmacy, cognitive decline, and impairment of kidney and liver functions. Therefore, cautious initiation of HF treatment with a close follow-up of the blood pressure, liver functions, kidney functions, and electrolytes are of utmost importance.

## Introduction and background

Population aging is recognized as one of the significant demographic trends due to developments in medical research and general improvements in public health, resulting in a larger share of the population that is 65 and up. By 2050, an estimated 426 million people will be 80 or older. This number is projected to triple from its 2020 level [1].

Due to advances in cardiac interventional and imaging techniques and better-targeted medications, the elderly population now has a higher prevalence of cardiac comorbidities, hypertension, and other risk factors for heart failure (HF). Age-adjusted mortality rates and HF prevalence have decreased due to better treatment of cardiovascular disease in industrialized nations. On the other hand, the aging population means that HF is becoming more common [2-5]. Those over 70 have a tenfold higher HF prevalence than those under 55. Fifty percent of all HF patients have heart failure with reduced ejection fraction (HFrEF, i.e., left ventricular ejection fraction (LVEF) <40%), with the other fifty percent having heart failure with preserved ejection fraction (HFpEF, i.e., LVEF ≥ 50%) or heart failure with mildly reduced ejection fraction (HFmrEF, i.e., LVEF=40%-49%) [6-8].

Common causes of HFpEF in the elderly include CAD, hypertension, age-related degenerative valvular disease, arrhythmias such as atrial fibrillation (AF), cardiac amyloidosis, and pericardial diseases such as constrictive pericarditis [9]. The MAGGIC meta-analysis concluded that HFpEF has lower age-adjusted mortality than HFrEF [10]. 30% of the 30-day rehospitalizations in HF patients were for heart failure [11]. A subsection of patients with HFpEF is found to have declining EF on subsequent follow-ups, which carries a much worse prognosis than HFrEF [12].

**Figure 1:**
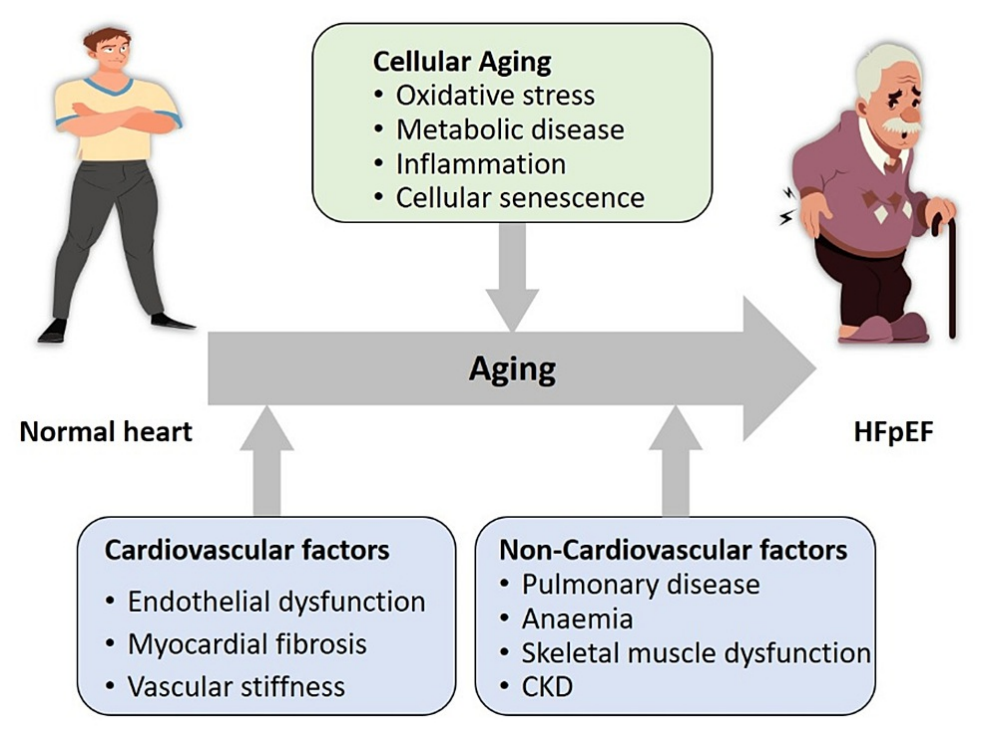
The interaction between age and other triggering factors to produce HFpEF. Adapted from Sauer et al. [14]. The figure was designed by the author Amr Elkammash.

The causes and effects of HFpEF have been the focus of numerous scientific investigations. In addition to preexisting risk factors, aging promotes myocardial remodeling and microvascular endothelial dysfunction, both of which can contribute to Left ventricular (LV) impairment and the development of HF. LV hypertrophy, chronotropic intolerance, and the acceleration of inflammatory pathways in the pathogenesis of heart failure can be triggered by risk factors such as obesity, hypertension, diabetes, and myocardial ischemia (Figure 1) [13].

It can be difficult to determine the pathophysiological mechanisms underlying HFpEF, especially in individuals with several risk factors, which makes it difficult to identify and optimize the proper treatment [12,15]. In addition, elderly patients are generally at higher risk of side effects from HF medications due to a higher prevalence of polypharmacy, cognitive impairment, and reduced liver and kidney functions [16]. In this review, we tried to illustrate the safety and effectiveness of different modalities to manage HFpEF in the elderly.

Methodology

**Figure 2:**
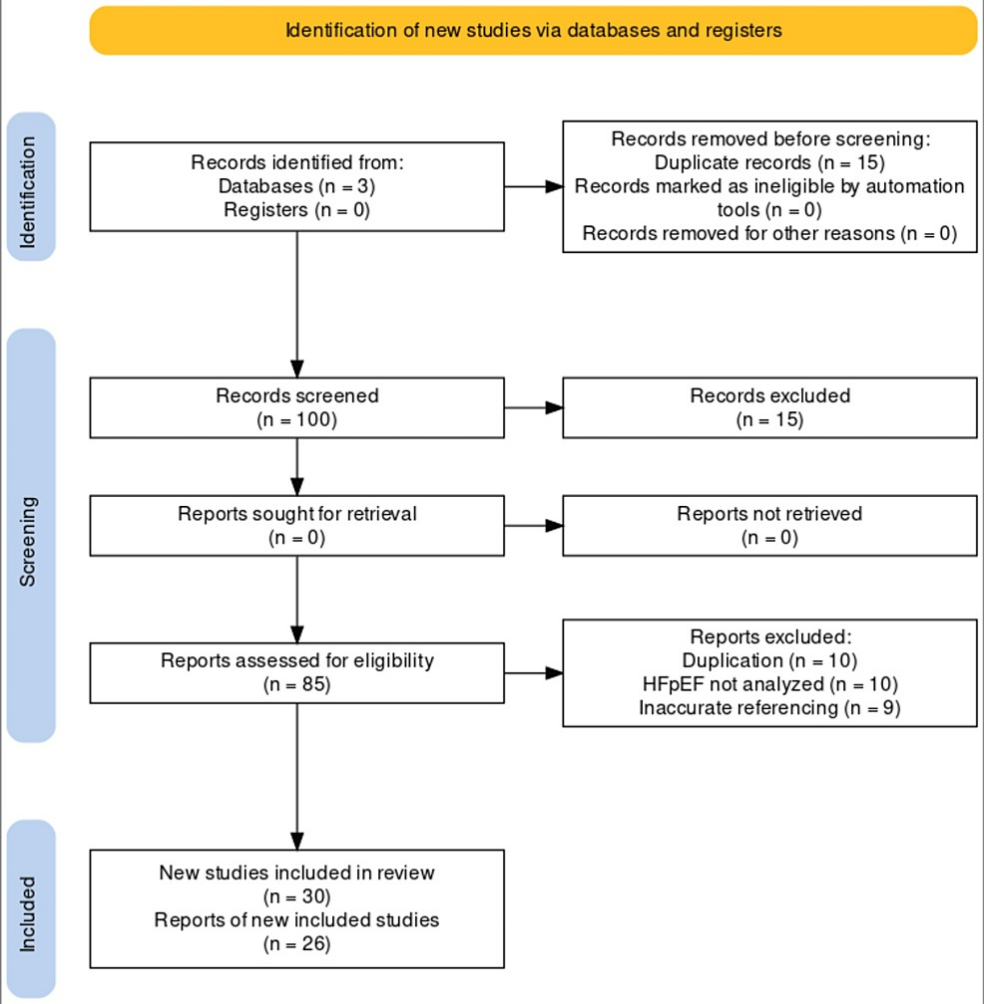
PRISMA flowchart summarizing the methodology of the review.

We searched the largest three scientific databases ( Google Scholar, Pubmed, EMBASE) using the MeSH terms and keywords (elderly, HFpEF, heart failure with preserved ejection fraction, guidelines, treatment, management) in different combinations. We retrieved 100 records initially and excluded 15 of them due to duplication. We then screened the remaining records and excluded another 29 records for the following reasons: 10 records due to the duplication of information, ten records where the HFpEF patients category was not analyzed, and nine records with inaccurate referencing (a reference to nowhere). We included the remaining 56 reports in a narrative review to indicate the latest evidence on managing HFpEF in the elderly population. The methodology is summarized in Figure 2 using the PRISMA flowchart [17].

## Review

Management of HFpEF in the elderly

The 2021 ESC guidelines on the management of HF recommended that a multidisciplinary team should manage HF patients, including an HF specialist cardiologist, an HF nurse, a geriatrician, a dietician, a psychologist, a physiotherapist, and an occupational therapist, with other specialist input according to the patient's needs [12].

Control of the Causes and Risk Factors of HFpEF

Most HF-specific treatments have not significantly reduced the primary endpoints of CV morbidity and mortality in randomized controlled trials (RCTs). Therefore, the 2021 European Society of Cardiology (ESC) HF guidelines recommended controlling the causes and risk factors of HFpEF as the main line of treatment for this problem [12]. The important causes to be targeted include hypertension [18], cardiac amyloidosis [19], AF [20], valvular diseases, and CAD [12]. Weight reduction in obese patients can improve symptom control [21].

HF-Specific Therapies

These included non-pharmacological and pharmacological approaches.

Non-pharmacological Approaches

Exercise training: Even among the elderly, exercise training has been shown to enhance peak and submaximal exercise capacity, 6-minute walking distance, and overall quality of life compared to non-pharmacological treatments. Supervised exercise training (either resistance or endurance) was shown to improve exercise capacity (peak VO2) and reverse remodeling of the atrium and left ventricular diastolic dysfunction in all patients above and above the benefits gained from standard medical therapy [22]. In the REHAB-HF trial, Kitzman et al. [23] demonstrated the benefit of a supervised exercise program in improving the functional capacity of elderly patients after admission for acute HF, including HFpEF patients.

Device therapy: Hospitalizations for heart failure (including HFpEF) were significantly reduced using the wireless implantable hemodynamic monitoring system (CardioMEMS) of the pulmonary artery pressure [24,25]. The benefit extended over a mean follow-up period of 13 months [26]. Another emerging device is the interatrial shunt device. It is a percutaneously inserted interatrial conduit that aims to decompress the left side of the heart, reducing the left atrial pressure and the pulmonary capillary wedge pressure whenever they are high [27]. The evidence on this device is still controversial without a significant improvement in cardiovascular morbidity and mortality [28,29].

Pharmacological Approaches

The main trials that studied the pharmacological treatment of HFpEF are summarized in Table 1.

**Table 1:** The main trials that studied the pharmacological management of HFpEF. Age is expressed in mean±SD or median (IQR). ↓: reduction. KCCQ-CS: Kansas City Cardiomyopathy Questionnaire Clinical Summary Score. CV: cardiovascular. QoL; quality of life. MRA: Mineralocorticoid receptor antagonists. BBs: Beta-blockers. ACEIs: Angiotensin-converting enzyme inhibitors. ARBs: Angiotensin receptor blockers. SGLT2: Sodium-Glucose Cotransporter 2.

Trial	Intervention arms	Age of the participants	Main outcomes
MRAs:			
ALDO-DHF [30]	Spironolactone vs. placebo	67±8 years	Improvement of the diastolic function but not the HF symptoms or QoL.
BBs:			
SENIORS [31]	Nebivelol vs. placebo	≥70 years	↓ all-cause mortality or CV hospitalization
ELANDD [32]	Nebivelol vs. placebo	66.5±9.8 years	No significant improvement in exercise capacity.
J-DHF [33]	Carvidelol vs. placebo	73±10 years	No significant improvement in CV mortality and unplanned HF hospitalizations.
ACEIs:			
PEP-CHF [34]	Perindopril vs. placebo	≥70 years	? improved HF symptoms and exercise capacity and reduced HF hospitalizations (underpowered)
Kitzman et al. [35]	Enalapril vs. placebo	70 ±1 years	No significant improvement in exercise capacity and aortic distensibility.
ARBs:			
CHARM-PRESERVED [36]	Candesartan vs. placebo	Mean age 65 years ( 601 patients aged ≥ 75 years)	↓HF hospitalizations, no CV mortality reduction.
I-PRESERVE [37]	Irbesartan vs. placebo	72±7 years	No significant improvement in all-cause mortality or CV hospitalizations.
Hong Kong diastolic heart failure study [38]	diuretic, diuretic + Ramipril, diuretic + Irbesartan	≥ 65 years	Only diuretics improved HF symptoms. Diuretic +ACEIor ARB improved LVsystolic and diastolic longitudinal function.
Digoxin:			
DIG-PEF [39]	Digoxin vs. placebo	66.7±10.7 years	↓HF hospitalizations but no CV mortality reduction
SGLT2 inhibitors:			
EMPEROR-PRESERVED [40]	Empagliflozin vs. placebo	71.8±9.3 years	↓CV death or HF hospitalizations
PRESERVED-HF [41]	Dapagliflozin vs. placebo	Median age 69 years (64,77)	Improvement of the KCCQ-CS score
DELIVER [42]	Dapagliflozin vs. placebo	71.8±9.6 years	↓CV death or worsening of HF
SCORED [43]	Sotagliflozin vs. placebo in T2DM with CKD patients	Median age 69 years (63,74)	↓ CV mortality, HF hospitalizations, urgent visits for HF
SOLOIST-WHF [44]	Sotagliflozin vs. placebo in T2DM without CKD patients	Median age 69 years (63,76)	↓ CV mortality, HF hospitalizations, urgent visits for HF
Sacubitril/Valsartan:			
PARAGON-HF [45]	Sacubitiril/Valsartan vs. Valsartan	72.7±8.3 years	No significant reduction of CV death or HF hospitalizations.

Diuretics

Loop diuretics: Loop diuretics can only control the symptoms of fluid overload in HFpEF patients; however, the evidence of their effect on CV hospitalizations and mortality is controversial [46]. Montero et al. [47] showed that elderly HFpEF patients treated with loop diuretics had impaired VO2 and diastolic function that is linearly related to the dose of the diuretic. Parajuli et al. [48] showed that HFpEF patients on loop diuretics had a lower 30-day HF readmission rate, independent of other HF medications.

Mineralocorticoid receptor antagonists (MRAs): Similar to loop diuretics, the evidence on the usefulness of MRAs in the elderly with HFpEF is inconsistent. Edelmann et al. [30] showed that Spironolactone improved the echocardiographic diastolic function in HFpEF patients without improvement of HF symptoms or quality of life. Eplerenone showed the same later effects in the RAAM-PEF trial [49]. Patel et al. [50] demonstrated that MRAs did not affect the outcomes in older HFpEF patients. A meta-analysis of the three landmark trials on MRAs (TOPCAT, EMPHASIS-HF, and RALES) showed that MRAs reduced morbidity and mortality in elderly patients with both HFrEF and HFpEF [51].

The use of diuretics in the elderly is generally associated with a higher incidence of adverse drug effects, including orthostatic hypotension, dehydration, renal impairment, and electrolyte disturbances. Clinicians should take care of regular monitoring of renal functions and electrolytes, patient education on orthostatic hypotension, and start the diuretics at low doses with slow titration to the desired doses [16].

Beta-Blockers (BBs)

Despite BBs' well-established benefit in treating HFrEF [52], their benefit in treating patients with HFpEF is debatable. BBs are commonly used to manage CAD and arrhythmias in HFpEF patients [12]. HFpEF patients may not increase their heart rate sufficiently during exercise, leading to exercise intolerance. By slowing the heart rate, BBs might worsen such chronotropic incompetence and exacerbate their symptoms [53].

Van Veldhuisen et al. [31] showed Nebivelol has significantly reduced the primary endpoint of all-cause mortality or CV hospitalization in elderly HFpEF patients in a subanalysis of the SENIORS trial. Fukuta et al. [53] conducted a meta-analysis which suggested that BBs may improve heart failure symptoms in HFpEF patients with CAD or AF, while those with neither CAD nor AF may not receive the potential benefit of BBs. On the other hand, several other trials did not show the benefit of BBs in improving cardiovascular outcomes and QoL in elderly HFpEF patients. In the OPTIMIZE-HF registry analysis, BBs generally failed to improve HF hospitalizations and mortality in HFpEF patients, including the elderly age group [54]. Similarly, Carvidelol did not improve the composite primary endpoint of cardiovascular mortality and unplanned hospitalization for HF in elderly HFpEF patients in the J-DHF trial [33]. Nebivolol also did not improve the exercise performance of those patients in the ELANDD study after six months of treatment [32].

The risk of symptomatic bradycardia is higher in elderly patients on BBs. The penetrance of the lipid-soluble BBs (e.g., Metoprolol) is more common in the elderly, resulting in central nervous side effects (e.g., fatigue). Switching the patient to non-lipid-soluble BBs helps reduce the incidence of the latter [16].

Renin-Angiotensin-Aldosterone System (RAAS) inhibitors:

Angiotensin-converting enzyme inhibitors (ACEIs): Cleland et al. [34] suggested in the PEP-CHF trial that using Perindopril in elderly HFpEF patients improved HF symptoms and exercise capacity and reduced HF hospitalizations. However, the study was underpowered to confirm such benefits. This study was followed by another study where Kitzman et al. [35] failed to show the improvement of exercise tolerance and aortic distensibility in the elderly with HFpEF using Enalapril.

Angiotensin-receptor blockers (ARBs): The CHARM-PRESERVED trial was a double-blinded placebo-controlled RCT that showed an improvement in the rate of HF hospitalization in HFpEF patients using Candesartan; however, the CV mortality in those patients did not improve [36]. On the contrary, the I-PRESERVE trial did not show the advantage of Irbesartan in elderly HFpEF patients in terms of reduction in the primary outcomes (all-cause mortality and hospitalization due to cardiovascular causes) and the secondary outcomes ( the improvement of heart failure symptoms or hospitalization due to heart failure) [37].

Yip et al. [38] assessed the effects of diuretics, Ramipril, or Irbesartan in combination with diuretics on HF symptoms' improvement in HFpEF patients. The study revealed a lack of significant difference in HF symptoms in the three treatment groups. Such lack of benefit could point to the fact that symptoms in HFpEF patients are likely due to fluid overload, and ACE inhibitors and ARBs have little to no role to play in symptom improvement.

Elderly patients are more susceptible to developing side effects from the use of RAAS inhibitors, including postural hypotension, kidney failure, and electrolyte derangement. Good patient counseling, regular monitoring of kidney functions and electrolytes, and slow dose titration can help mitigate these adverse effects [16]. These side effects also occur with angiotensin-receptor-Neptrilysin inhibitors (ARNIs) [55].

Digoxin

In the Digitalis Interaction Group (DIG) trial, 988 patients with an EF of more than 45% were randomly assigned to receive either a placebo or digoxin [39]. Despite encouraging trends toward less hospitalization and greater exercise tolerance, no substantial decreases in deaths due to HF were observed.

Sodium-Glucose Cotransporter 2 (SGLT-2) Inhibitors

The 2022 AHA/ACC/HFSA guidelines for the management of heart failure recommended using SGLT2 inhibitors (Class IIa recommendation) for treating patients with HFpEF [56]. 

Empagliflozin proved its effectiveness in reducing the composite endpoint of cardiovascular death or hospitalization for HF in HFpEF patients across different age groups in the EMPEROR-Preserved RCT, irrespective of the diabetic state [40]. The age group of 80 years and above included 1,299 patients. The proportion of atrial arrhythmias and the prevalence of New York Heart Association functional class III symptoms were highest in this elderly population. LVEF was best preserved, N-terminal pro-B-type natriuretic peptide was highest, and eGFR was lowest in that age group. There was no discernible difference in the occurrence of serious adverse events leading to medication discontinuation between the treatment and placebo groups across the various age ranges studied.

Another SGLT2 inhibitor, Dapagliflozin, was assessed in two RCTs on HFpEF patients, the PRESERVED-HF [41] and the DELIVER [42] trials. In the PRESERVED-HF trial, Dapagliflozin significantly improved the score of HFpEF patients on the Kansas City Cardiomyopathy Questionnaire Clinical Summary Score (KCCQ-CS) across different age groups, including those above 70 years of age [41]. In the DELIVER trial, Dapagliflozin reduced the composite primary outcome of worsening heart failure (defined as either an unplanned hospitalization for heart failure or an urgent visit for heart failure) or cardiovascular death in HFpEF patients above 72 years of age. Dapagliflozin was beneficial irrespective of their diabetic state and without an increase in adverse effects [42].

Bhatt et al. tested Sotagliflozin in two types of HFpEF patients with type 2 diabetes mellitus (T2DM): those with chronic kidney disease (in the SCORED trial [43]) and those without (in the SOLOIST-WHF trial [44]). The median age in both trials was 69 years. The Sotagliflozin effectively reduced the primary outcomes of CV death, HF hospitalizations, and urgent visits for HF.

Risks of the use of SGLT2 inhibitors in the elderly includes increased genitourinary infections, hypoglycemia, volume depletion, bone fractures, and euglycemic diabetic ketosis. However, the risks are not increased in the elderly compared to other age groups, and the benefits of reduced CV death, HF hospitalizations, and renal adverse outcomes outweigh the risks [57].

Angiotensin Receptor-Neprilysin inhibitor (ARNI)

The PARAGON-HF was a large RCT that included 4822 HFpEF patients (46% aged 75 years or older). In that study, there was no significant difference between the ARNI Sacubitril-Valsartan group and the Valsartan groups in the composite primary endpoint of HF hospitalization or CV death. Nevertheless, the Sacubitril-valsartan group had a higher incidence of hypotension and a lower incidence of renal impairment and hyperkalemia than the Valsartan group [45].

## Conclusions

Treatment of HFpEF remains a significant challenge in managing HF patients, especially in the elderly. The management should focus on addressing the causes of HFpEF and the associated comorbidities in the affected individuals. To date, SGLT2 inhibitors are the only agents proven by randomized clinical trials to effectively reduce the hard endpoints of CV mortality and HF hospitalizations. Exercise training and loop diuretics can improve HF symptoms without a clear benefit on CV mortality and HF hospitalizations. The benefit of the other HF disease-modifying agents in HFpEF is controversial. Elderly HFpEF patients are susceptible to developing adverse effects from HF medications; therefore, very slow initiation of medications and close follow-up of the blood pressure, renal functions, liver functions, and electrolytes are mandatory. Adjustments in the prescriptions should be considered on a case-to-case basis.
